# Diagnostic values and appropriate cutoff points of lipid ratios in patients with abnormal glucose tolerance status: a cross-sectional study

**DOI:** 10.1186/s12944-019-1070-z

**Published:** 2019-06-01

**Authors:** Wen Guo, Pei Qin, Jing Lu, Xiaona Li, Wenfang Zhu, Nianzhen Xu, Jianming Wang, Qun Zhang

**Affiliations:** 1 Department of Health Promotion Center, The First Affiliated Hospital with Nanjing Medical University, 300 Guangzhou Road, Nanjing, 210029 China; 2 School of Public Health, Nanjing Medical University, 818 Tianyuan East Road, Nanjing, 211166 China

**Keywords:** Lipid ratio, Dyslipidemia, Glucose tolerance, Diabetes

## Abstract

**Background:**

Lipid ratios, for example total cholesterol/high-density lipoprotein cholesterol (TC/HDL-C) and triglyceride/high-density lipoprotein cholesterol (TG/HDL-C), are associated with type 2 diabetes mellitus (T2DM). However, the predictive values of lipid ratios in prediabetes remain unclear. The aims of this study were: 1) to investigate the association between lipid ratios and abnormal glucose tolerance; 2) to compare the predictive significance of lipid ratios with commonly used indicators of lipid variables in clinical practice in a Chinese population.

**Methods:**

The cross-sectional study enrolled 2680 participants from the Health Promotion Center of the First Affiliated Hospital of Nanjing Medical University. All participants received a 75 g oral glucose tolerance test. Blood samples were obtained at baseline and 120 min after glucose ingestion. Participants were classified as normal glucose tolerance (NGT), impaired glucose regulation (IGR), and T2DM. The odds ratios (ORs) and 95% confidence intervals (CIs) were estimated using logistic regression model. The receiver operating characteristic (ROC) curve was used to identify the cutoff points of lipid and lipid ratios. The area under the receiver operating characteristic curve (AUROC), sensitivity and specificity were calculated to estimate their diagnostic values.

**Results:**

TC, TG, TC/HDL-C, TG/HDL-C and non-HDL-C were significantly correlated with both prediabetes and T2DM after adjustment for other risk factors such as blood glucose, whereas LDL-C was only positively correlated with prediabetes. TG and TG/HDL-C showed higher diagnostic values for prediabetes and T2DM than TC, LDL-C, HDL-C, TC/HDL-C and non-HDL-C, with the AUC values over 0.70. For predicting prediabetes, the optimal cutoff point was 1.36 mmol/l for TG and 1.13 for TG/HDL-C. For predicting T2DM, the optimal cutoff point was 1.46 mmol/l for TG and 1.22 for TG/HDL-C.

**Conclusions:**

Both TG and TG/HDL-C are promising biomarkers for distinguishing individuals with abnormal glucose tolerance, and can be used to predict prediabetes and T2DM in Chinese population.

## Background

The prevalence of diabetes has increased significantly over recent decades in China. According to the latest national survey of 98,658 adults from 31 provinces (autonomous regions and municipalities) in mainland China in 2010, the prevalence rate was 11.6% for diabetes, 3.5% for isolated post-challenge hyperglycemia (IPH), and 8.4% for impaired glucose tolerance (IGT). In this survey, IPH was defined as fasting plasma glucose (FPG) < 7 mmol/l and 2-h post-challenge plasma glucose (2-hPG) ≥11.1 mmol/l, and IGT was defined as FPG < 6 mmol/l and 2-hPG ≥7.8 mmol/l. [1] People with IPH or IGT have similar risk of target organs damage as people with diabetes [2, 3], but this population are often ignored and untreated owing to the high cost of a traditional oral glucose tolerance test (OGTT). Thus, an effective method for the early detection of patients with IPH or IGT is crucial for effective intervention and therapy.

Type 2 diabetes mellitus (T2DM) is the most common type of diabetes. Changes in lifestyle, including “nutrition transition” and physical inactivity, increase the risk of diabetes. T2DM is often related to and accompanied by aberrant levels of plasma triglycerides (TG), total cholesterol (TC), high-density lipoprotein cholesterol (HDL-C), and low-density lipoprotein cholesterol (LDL-C) [4]. In the past few years, the lipid ratios, such as TC/HDL-C and TG/HDL-C, have been proposed as alternative biomarkers for predicting T2DM, because they can provide a set of integrated information based on multiple variables. For example, a prospective study reported that lipid ratios of TG/HDL-C and TC/HDL-C were associated with the incidence of T2DM among Iranians [5]. Another cross-sectional study in a Chinese community population found that TC/HDL-C was significantly related to T2DM and superior to LDL-C or HDL-C as a biomarker of diabetes risk [6]. However, there are limited data available about the role of lipid ratios in prediabetes.

The aim of this study was to investigate the association between lipid ratios and abnormal glucose tolerance, including prediabetes and T2DM. In addition, this study compared the predictive significance of lipid ratios and traditional lipid indicators, such as TC, LDL-C, TG and HDL-C.

## Methods

### Subjects

We performed a cross-sectional study by enrolling 2680 participants from the Health Promotion Center of the First Affiliated Hospital of Nanjing Medical University. All participants underwent a 75 g OGTT. Blood samples were collected at baseline and 120 min after glucose ingestion. According to the World Health Organization (WHO) diagnostic criteria, 1027 participants were classified as having normal glucose tolerance (NGT), 834 participants were classified as having impaired glucose regulation (IGR), and 819 participants were classified as having T2DM. All study participants signed a written consent form. Participants with the following characteristics were excluded: diabetic ketosis, hyperthyroidism, liver disorders, kidney disorders and other diseases associated with lipid metabolism dysfunction, or consuming statin lipid-lowering drugs. This study was approved by the Ethical Committee of the First Affiliated Hospital of Nanjing Medical University (2016-SR-220).

### Blood sample collection and storage

All participants underwent an oral glucose tolerance test (OGTT) after overnight fasting. Blood samples were collected before and 120 min after the glucose load, and centrifuged at 4 °C (1,610 g/min for 15 min). Serum samples were separated and stored at − 80 °C.

### Physical examination and biochemical tests

Weight, height and blood pressure (BP) were measured in accordance with international standards. Plasma levels of glucose, TC, TG, LDL-C and HDL-C concentrations were measured by enzymatic methods (Chemistry Analyzer Au2700, Olympus Medical Engineering Company, Japan). Non-HDL-C was calculated by subtracting HDL-C from TC. The TC/HDL-C and TG/HDL-C ratios were calculated by dividing TC and TG by HDL-C.

### Statistical analysis

Continuous variables were expressed as mean ± SD. Differences among groups were tested by one-way ANOVA with Bonferroni correction for pairwise comparisons. The logistic regression model was used to examine the association between glucose status and lipid parameters, and was adjusted for potential confounders such as age, sex, BP, smoking status and BMI. The odds ratios (ORs) and 95% confidence intervals (95% CIs) were used to estimate the strength of association. The diagnostic property of lipid parameters in IGR or T2DM was evaluated using the area under the curve (AUC) of the receiver operating characteristics (ROC). Data were analyzed using SPSS18.0 statistical software, with significance defined as *p* < 0.05 (two-sided).

## Results

### Participant’s characteristics

Clinical and biochemical characteristics of the participants are listed in Table 1. There were 1027 participants with NGT, 834 with IGR and 819 with newly diagnosed T2DM. Participants with IGR and T2DM were characterized by increasingly older age, higher blood pressure and BMI, greater levels of LDL-C, TC, TG, TC/HDL-C, TG/HDL-C, non-HDL-C, FBG and 2-hPG and lower levels of HDL-C, compared with participants with NGT (*P* < 0.05).

**Table 1:** The clinical and biochemical properties of 2680 participants

	NGT (*n* = 1027)	IGR(*n* = 834)	T2DM(*n* = 819)
Age(years)	51.81 ± 10.59	57.79 ± 9.19^b^	60.07 ± 9.17^bd^
Male/female	415/612	379/455	344/475
Smoking (%)	225(21.91)	269(32.25) ^b^	360(43.95)^b,d^
SBP(mmHg)	125.57 ± 17.60	136.64 ± 18.45^b^	141.42 ± 19.44^b,d^
DBP((mmHg)	77.26 ± 11.00	81.21 ± 11.15^b^	82.88 ± 11.55^b^
BMI(kg/m^2^)	23.68 ± 3.01	25.82 ± 2.98^b^	26.18 ± 3.10^b,c^
HDL-C(mmol/l)	1.35 ± 0.31	1.22 ± 0.23^b^	1.21 ± 0.24^b^
LDL-C(mmol/l)	2.88 ± 0.63	3.12 ± 0.81^b^	3.15 ± 0.79^b^
TC(mmol/l)	4.78 ± 0.82	5.21 ± 0.97^b^	5.32 ± 1.02^b^
TG(mmol/l)	1.23 ± 0.56	2.05 ± 0.92^b^	2.44 ± 1.76^b,d^
TC/HDL-C	3.65 ± 0.78	4.34 ± 0.83^b^	4.49 ± 1.01^b,d^
TG/HDL-C	0.98 ± 0.56	1.74 ± 0.90 ^b^	2.20 ± 2.28^b,d^
non-HDL-C	3.43 ± 0.74	3.98 ± 0.88 ^b^	4.10 ± 0.93^b,c^
FBG(mmol/l)	5.21 ± 0.41	5.86 ± 0.55^b^	7.44 ± 2.08^b,d^
2-hPG(mmol/l)	5.89 ± 1.04	8.61 ± 1.35^b^	14.31 ± 3.94^b,d^

Abbreviations: *SBP* systolic blood pressure, *DBP* diastolic blood pressure, *BMI* body mass index, *HDL-C* high-density lipoprotein cholesterol, *LDL-C* low-density lipoprotein cholesterol, *TC* total cholesterol, *TG* triglyceride, *FBG* fasting blood glucose, *2-hPG* 2-h post challenge plasma glucose, Compared with NGT, ^a^*P* < 0.05, ^b^*P* < 0.01; Compared with IGR, ^c^*P* < 0.05, ^d^*P* < 0.01

### Multinomial logistic regression analysis for the risk of IGR and T2DM

After adjusting for age, sex, blood pressure smoking status, BMI, FBG and 2-hPG, the LDL-C, TC, TG, TC/HDL-C, TG/HDL-C and non-HDL-C were all positively correlated with IGR, with the OR (95% CI) of 1.532(1.178–1.991) for LDL-C (one mmol/l increase), 1.660 (1.341–2.055) for TC (one mmol/l increase), 3.954 (2.739–5.597) for TG (one mmol/l increase), 2.041 (1.566–2.661) for TC/HDL-C (per unit increase), 3.445 (2.417–4.921) for TG/HDL-C (per unit increase), 1.970 (1.548–2.506) for non-HDL-C (per unit increase), respectively (Table 2).

**Table 2:** Multinomial logistic analysis of the risk factors for type 2 diabetes

Variables	Model	IGR		T2DM	
		OR(95%CI)	P	OR(95%CI)	*P*
HDL-C(mmol/l)	1	0.168(0.118–0.240)	< 0.001	0.142(0.099–0.204)	< 0.001
	2	0.612(0.282–1.332)	0.216	0.763(0.213–2.737)	0.678
LDL-C(mmol/l)	1	1.571(1.382–1.785)	< 0.001	1.675(1.473–1.904)	< 0.001
	2	1.532(1.178–1.991)	0.001	1.299(0.881–1.915)	0.187
TC(mmol/l)	1	1.690(1.522–1.877)	< 0.001	1.888(1.698–2.100)	< 0.001
	2	1.660(1.341–2.055)	< 0.001	1.581(1.149–2.176)	0.005
TG(mmol/l)	1	7.283(6.005–8.833)	< 0.001	9.122(7.503–11.09)	< 0.001
	2	3.954(2.739–5.597)	< 0.001	4.677(3.128–6.993)	< 0.001
TC/HDL-C	1	3.133(2.743–3.578)	< 0.001	3.731(3.255–4.275)	< 0.001
	2	2.041(1.566–2.661)	< 0.001	1.762(1.225–2.536)	0.002
TG/HDL-C	1	6.326(5.244–7.630)	< 0.001	7.856(6.498–9.498)	< 0.001
	2	3.445(2.417–4.912)	< 0.001	3.943(2.625–5.923)	< 0.001
non-HDL-C	1	2.397(2.118–2.713)	< 0.001	2.751(2.426–3.120)	< 0.001
	2	1.970(1.548–2.506)	< 0.001	1.828(1.280–2.612)	0.001

Model 1: unadjusted

Model 2: adjustment for BP, sex, smoking, age, BMI, FBG and 2-hPG

TC, TG, TC/HDL-C, TG/HDL-C and non-HDL-C were all positively correlated with T2DM, with the OR (95% CI) of 1.581(1.149–2.176) for TC (one mmol/l increase), 4.677 (3.128–6.993) for TG (one mmol/l increase), 1.762 (1.225–2.536) for TC/HDL-C (per unit increase), 3.943 (2.625–5.923) for TG/HDL-C (per unit increase), and 1.828 (1.280–2.612) for non-HDL-C (per unit increase) (Table 2).

### Binary logistic regression analysis on the factors related with IGT or IPH

There were 691 participants with normal FBG but abnormal 2-hPG who were therefore defined as IGT or IPH. After adjusting for age, sex, blood pressure, smoking, BMI and FBG, logistic regression analysis revealed that HDL-C (*OR*: 0.229, 95% *CI*: 0.184–0.486), LDL-C (*OR*: 1.584, 95% *CI*: 1.338–1.873), TC (*OR*: 1.691, 95% *CI*: 1.473–1.943), TG (*OR*: 6.221, 95% *CI*: 4.841–7.933), TC/HDL-C (*OR*: 2.680, 95% *CI*: 2.242–3.204), TG/HDL-C (*OR*: 5.535, 95% *CI*: 4.311–7.108) and non-HDL-C (*OR*: 2.180, 95% *CI*: 1.857–2.559) were significantly associated with IGT or IPH (Table 3).

**Table 3:** Multinomial logistic analysis of the risk factors for IGT or IPH

Variables	Model	IGT or IPH	
		OR(95%CI)	*P*
HDL-C(mmol/l)	1	0.162(0.109–0.239)	0.003
	2	0.229(0.184–0.486)	< 0.001
LDL-C(mmol/l)	1	1.561(1.348–1.808)	< 0.001
	2	1.584(1.338–1.873)	< 0.001
TC(mmol/l)	1	1.722(1.526–1.942)	< 0.001
	2	1.691(1.473–1.943)	< 0.001
TG(mmol/l)	1	7.921(6.303–9.953)	< 0.001
	2	6.221(4.841–7.933)	< 0.001
TC/HDL-C	1	3.205(2.748–3.738)	< 0.001
	2	2.680(2.242–3.204)	< 0.001
TG/HDL-C	1	6.732(5.411–8.376)	< 0.001
	2	5.535(4.311–7.108)	< 0.001
non-HDL-C	1	2.475(2.145–2.856)	< 0.001
	2	2.180(1.857–2.559)	< 0.001

Model 1: unadjusted

Model 2: BP, sex, smoking, age, BMI, FBG and 2-hPG

### Diagnostic value of lipid parameters for T2DM

**Fig. 1:**
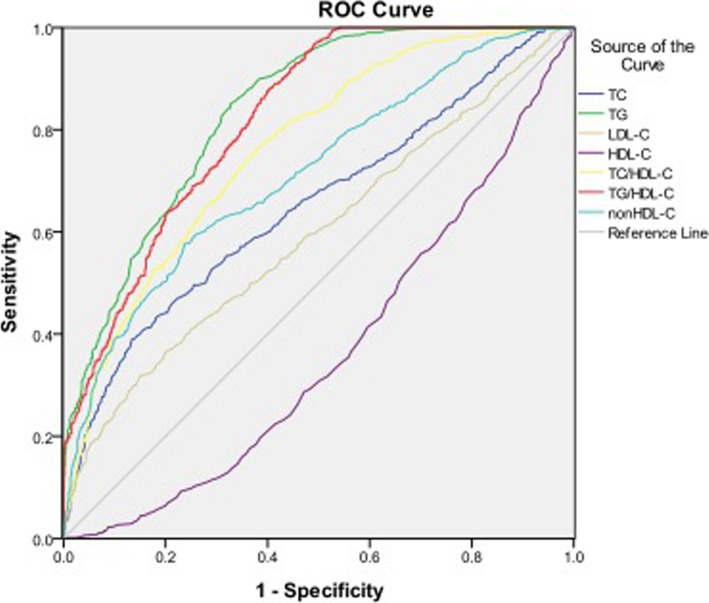
Area under the receiver operating characteristics curves (AUROCs) of lipid markers for type 2 diabetes

Table 4 and Fig. 1 showed the cutoff points of lipid parameters for the prediction of T2DM with their corresponding specificity and sensitivity. The AUROCs for TG, TC/HDL-C, TG /HDL-C and non-HDL-C were all > 0.7, indicating they are potential predictors of T2DM. Both TG and TG/HDL-C had the AUROC> 0.8. The optimal cutoff points of TG, TC/HDL-C, TG /HDL-C and non-HDL-C for predicting T2DM were 1.46 mmol/l, 3.92, 1.22 and 3.76, respectively.

**Table 4:** ROC curve for predicting type 2 diabetes and cutoff points for maximum sum of sensitivity and specificity

	ROC (95%CI)	Cutoff point	Sensitivity(%)	Specificity(%)
HDL-C(mmol/l)	0.366(0.341–0.391)	1.29	55.80	64.70
LDL-C(mmol/l)	0.595(0.569–0.621)	3.05	51.80	60.70
TC(mmol/l)	0.651(0.626–0.676)	5.18	53.20	70.30
TG(mmol/l)	0.838(0.820–0.855)	1.46	78.50	71.40
TC/HDL-C	0.764(0.742–0.785)	3.92	72.30	65.50
TG/HDL-C	0.822(0.804–0.840)	1.22	72.30	71.40
non-HDL-C	0.715(0.691–0.738)	3.76	62.10	69.90

### Diagnostic value of lipid parameters for prediabetes

**Fig. 2:**
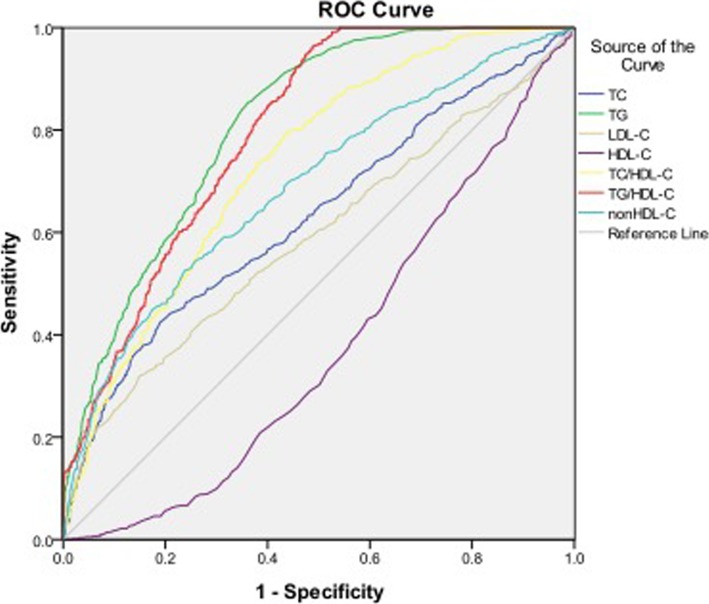
Area under the receiver operating characteristics curves (AUROCs) of lipid markers for prediabetes

As shown in Table 5 and Fig. 2, the AUROCs of TG, TC/HDL-C and TG /HDL-C were > 0.7. Similar to T2DM, TG and TG/HDL-C also showed the highest diagnostic value for prediabetes. The optimal cutoff points of TG, TC/HDL-C and TG /HDL-C for predicting prediabetes were 1.36 mmol/l, 3.83 and 1.13, respectively (Table 5).

**Table 5:** ROC curve for predicting prediabetes and cutoff points for maximum sum of sensitivity and specificity

	ROC (95%CI)	Cutoff point	Sensitivity (%)	Specificity (%)
HDL-C(mmol/l)	0.377(0.352–0.402)	1.30	54.60	64.00
LDL-C(mmol/l)	0.589(0.562–0.615)	3.07	52.30	61.80
TC(mmol/l)	0.631(0.605–0.656)	5.15	51.20	68.60
TG(mmol/l)	0.812(0.793–0.831)	1.36	80.20	67.40
TC/HDL-C	0.733(0.710–0.755)	3.83	73.70	61.80
TG/HDL-C	0.796(0.777–0.816)	1.13	73.50	67.50
non-HDL-C	0.689(0.665–0.713)	3.68	61.90	64.50

### Diagnostic value of lipid parameters for IGT or IPH

**Fig. 3:**
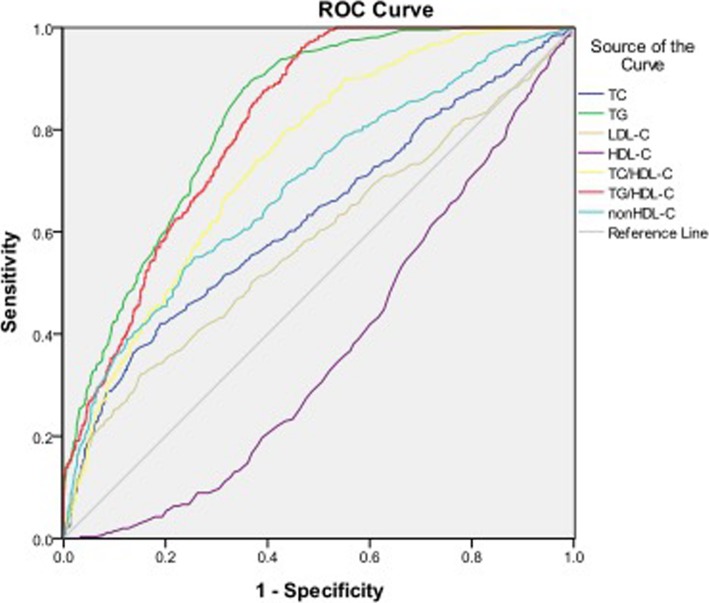
Area under the receiver operating characteristics curves (AUROCs) of lipid markers for IGT or IPH

The AUROCs of HDL-C, LDL-C, TC and non-HDL-C for IGT or IPH were < 0.7. Both TG and TG/HDL-C had the AUC > 0.8, showing their predictive values for IGT or IPH. The optimal cutoff points for TG, TC/HDL-C and TG/HDL-C were 1.37 mmol/l, 3.97 and 1.13, respectively (Table 6, Fig. 3).

**Table 6:** ROC curve for predicting IGT or IPH and cutoff points for maximum sum of sensitivity and specificity

	ROC (95%CI)	Cutoff point	Sensitivity (%)	Specificity (%)
HDL-C(mmol/l)	0.371(0.344–0.398)	1.29	56.20	63.30
LDL-C(mmol/l)	0.583(0.553–0.612)	3.07	51.30	61.60
TC(mmol/l)	0.629(0.600–0.658)	5.06	55.40	63.30
TG(mmol/l)	0.826(0.807–0.845)	1.37	82.40	68.50
TC/HDL-C	0.742(0.718–0.766)	3.97	67.40	67.90
TG/HDL-C	0.812(0.792–0.831)	1.13	75.90	68.30
non-HDL-C	0.689(0.663–0.716)	3.84	55.10	74.30

## Discussion

Early detection of prediabetes and T2DM is important for implementing early intervention strategies, yet the traditional OGTT is time-consuming and is difficult to carry out in the general population. Findings from this study show that both TG and TG/HDL-C have high diagnostic values for distinguishing individuals with abnormal glucose tolerance, indicating that these are promising biomarkers for screening individuals at high risk in the Chinese population.

Serum levels of TC, TG and HDL-C are commonly obtained biomarkers in physical examinations. A cross-sectional study in China found that TC/HDL-C was significantly related to T2DM [6]. In an Iranian cohort, TC/HDL-C and TG/HDL-C showed similar performance for diabetes prediction. However, HDL-C had a predictive effect for incident diabetes only among women [5]. Khaloo et al. reported that TC, log-transformed TG (Ln-TG), HDL-C, LDL-C, non-HDL-C, Ln-TG/HDL-C and TC/HDL-C were individually associated with the risk of T2DM. With multivariate adjustment for factors including fasting plasma glucose (FPG) change, HDL-C, ln-TG/HDL-C and TC/HDL-C remained significantly associated with T2DM risk [7]. Although these studies have documented the effect of dyslipidemia on T2DM, data on the association between lipid parameters and different levels of glucose tolerance are limited.

Increased levels of TG are associated with greater risk of diabetes [8]. In the current study, serum TG level was a strong predictor of prediabetes and T2DM, independent of the other risk factors. The AUROC of TG for predicting T2DM was > 0.8, with a sensitivity of 78.5% and a specificity of 71.4%. The AUROC of TG for predicting prediabetes was > 0.8, with a sensitivity of 80.2% and a specificity of 67.4%. Similar to our findings, a previous prospective study reported that TG is associated with the incidence of T2DM in an Iranian population, independent of other risk factors [5]. However, the AUROC of TG for predicting T2DM was smaller than in the current study (AUROC 0.5–0.7) and the TG cut off value is 1.98 mmol/l in men and 1.66 mmol/l in women, higher than in our study. The difference in TG levels between the studies might be due to lifestyle and baseline population differences.

Low HDL-C is known to be an important predictor of diabetes [9]. Certain agents known to raise HDL-C also improve glucose metabolism and prevent diabetes [10]. In our study, HDL-C was significantly negatively correlated with prediabetes and T2DM. However, after adjustment for other risk factors, HDL-C became non-significant, which was consistent with findings reported by Ley et al [11] Khaloo et al. found that a 1-SD change in HDL-C was significantly negatively associated with incident T2DM after adjustment for numerous confounders (HR: 0.84, 95% CI 0.76–0.93) [7]. Our study used baseline values of HDL-C rather than HDL-C change, which could contribute to the divergence between our findings and those of Khaloo et al.

Recent studies have indicated that the accumulation of cholesterol in β cells might contribute to lipotoxicity and β cell dysfunction [12]. A cross-sectional study provided further evidence that elevated serum levels of TC and LDL-C are associated with β cell dysfunction in participants with normal glucose tolerance [13]. Song et al. [6] showed that levels of TC and LDL-C were individually associated with T2DM, but the AUROC of TC and LDL-C for T2DM was small (AUROC 0.6–0.7) which is partly consistent with our results. In our study, we showed that TC and LDL-C were positively associated with prediabetes and T2DM, but after adjustment for other risk factors the association between LDL-C and T2DM was no longer statistically significant. The AUROC of TC and LDL-C for prediabetes and T2DM was also small (AUROC 0.5–0.7) in our study.

TG/HDL-C has been studied recently for potential clinical uses, including predicting atherosclerotic cardiovascular disease risk and micro- and macroangiopathies [14, 15]. TG/HDL-C is related to insulin resistance [16, 17] and β cell function, suggesting it might be a potential tool for identifying people with diabetes [18]. Two prospective studies suggested that high TG/HDL-C increased the risk of incident T2DM in the Chinese population [19, 20]. Another cross-sectional study in China suggested that TG/HDL-C was a better marker of insulin resistance and diabetes than routine lipid measures [21]. However, limited data were available regarding the association between TG/HDL-C and prediabetes. Our study found evidence that TG/HDL-C was a promising predictor for not only T2DM but also for prediabetes.

TC/HDL-C is an indirect estimate of LDL-C particle number and can be a strong predictor of the risk of atherosclerosis and coronary heart disease [22]. The role of TC/HDL-C in T2DM is still controversial. A cross-sectional study of 9078 Chinese individuals found that TC/HDL-C is superior to LDL-C and HDL-C levels at discriminating patients with T2DM [6]. A prospective study reported that TC/HDL-C and TG/HDL-C had similar performance for diabetes prediction in a population of Iranian men [5]. However, another study found that TC/HDL-C ratio was not a robust predictor of T2DM in high-risk individuals in Iran, with an AUROC of 0.55 [23]. Few studies have been performed to explore the relationship between TC/HDL-C ratio and prediabetes. In our study, although TC/HDL-C was positively correlated with prediabetes and T2DM, the diagnostic value was less than TG or TG/HDL-C.

Non-HDL cholesterol, including very-low-density lipoprotein (VLDL) remnant particles and intermediate density particles, is an indirect estimate of LDL particle number, and LDL particle number relates more closely to atherosclerosis and cardiovascular events than does LDL-C [24]. Liu et al. observed that non-HDL-C was superior to traditional cholesterol parameters in predicting incident diabetes in women but not in men [25]. Ley et al. reported that higher non-HDL-C cholesterol was associated with incident T2DM and was superior to LDL-C or HDL-C cholesterol for distinguishing individuals with and without incident diabetes in an Aboriginal Canadian population [26]. Liu et al. also found that levels of non-HDL-C were elevated in adults with prediabetes [27]. In our study, non-HDL-C was positively correlated with prediabetes and T2DM, with similar AUROCs.

Another major finding in our study is that TG and TG/HDL-C were associated with IGT and IPH. TG and TG/HDL-C were therefore potential markers for distinguishing individuals with normal FBG but abnormal 2 h-PG. Previous data showed that about half of individuals with undiagnosed diabetes in Asia met the criteria for diabetes on the basis of an elevated 2 h-PG after OGTT, despite their FBG being in the normal range [28]. The risk of developing diabetes was at least partly dependent on their post-load blood glucose level or glucose tolerance status [29, 30]. Individuals with IPH and IGT are often ignored and untreated owing to the higher cost and time-consuming nature of an OGTT; therefore, testing for TG and TG/HDL-C might be a convenient method to estimate levels of IPH and IGT. In our study, TG and TG/HDL-C were associated with IGT or IPH independently of other risk factors. Our study also found evidence that TG and TG/HDL-C were promising predictors of IGT or IPH. TG and TG/HDL-C are routine tests obtained during general physical examination. On the basis of these two commonly used indexes, high risk individuals can be rapidly identified and recommended to receive an OGTT.

Several limitations should be considered when interpreting the results of this study. First, the cross-sectional study design limits the ability to infer causality between lipid ratios and diabetes. Second, all participants were recruited from Jiangsu province, China. Therefore, it is uncertain whether these results are generalizable to other ethnic groups. Finally, the incidence of prediabetes and T2DM in individuals with TG and TG/HDL-C values above the cutoff values identified in this study requires exploration in a longitudinal study.

## Conclusions

This is the first study performed in a Chinese population demonstrating that TG and TG/HDL-C correlate not only with prediabetes and T2DM but also with IGT or IPH. This study shows the promising values of TG and TG/HDL-C are accessible biomarkers for distinguishing individuals with different levels of glucose tolerance, including those with normal FBG but abnormal 2-hPG.

## Data Availability

Data sharing is not applicable to this article as no data sets were generated or analyzed during the current study.

## Abbreviations

2-hPG: 2-h postchallenge plasma glucose
AUC: Area under the curve
BMI: Body mass index
CI: Confidence intervals
DBP: Diastolic blood pressure
FBG: Fasting blood glucose
HDL-C: High-density lipoprotein cholesterol
IGT: Impaired glucose tolerance
IPH: Isolated post-challenge hyperglycemia
LDL-C: Low-density lipoprotein cholesterol
NGT: Normal glucose tolerance
OGTT: Oral glucose tolerance test
OR: Odds ratio
ROC: Receiver operating characteristics
SBP: Systolic blood pressure
T2DM: Type 2 diabetes
TC: Total cholesterol
TG: triglyceride
